# Disseminated intravascular coagulation associated organ failure in obstetric patients admitted to intensive care units: a multicenter study in China

**DOI:** 10.1038/s41598-021-95841-7

**Published:** 2021-08-12

**Authors:** Zhiling Zhao, Jianxin Zhang, Nan Li, Gaiqi Yao, Yangyu Zhao, Shuangling Li, Qinggang Ge, Junli Lu, Shining Bo, Jingjing Xi, Yue Han

**Affiliations:** 1 Department of Intensive Care Unit, Peking University Third Hospital, 49 North Garden Rd., Haidian District, Beijing, 100191 People’s Republic of China; 2 Department of Gynecology and Obstetrics, Capital Medical University Affiliated Beijing Chao-Yang Hospital, Beijing, China; 3 Department of Research Center of Clinical Epidemiology, Peking University Third Hospital, Beijing, China; 4 Department of Gynecology and Obstetrics, Peking University Third Hospital, Beijing, China; 5 Department of Surgical Intensive Care Unit, Peking University First Hospital, Beijing, China; 6 Department of Intensive Care Unit, Capital Medical University Affiliated Beijing Chao-Yang Hospital, Beijing, China

**Keywords:** Diseases, Medical research, Coagulation system

## Abstract

We aimed to determine disseminated intravascular coagulation (DIC)-associated organ failure and underlying diseases based on data from three ICU wards in tertiary hospitals in China from 2008 to 2016. The diagnosis of DIC was confirmed by an International Society of Thrombosis and Hemostasis score greater than or equal to 5. The maternal outcomes included the changes in organ function 24 h after ICU admission. The durations of hospital stay and ICU stay were recorded as secondary outcomes. Among 297 ICU admissions (median Sequential Organ Failure Assessment score, 4) for obstetric diseases, there were 87 DIC cases, with an estimated DIC incidence of 87 per 87,580 deliveries. Postpartum hemorrhage was the leading disease associated with DIC (71, 81.6%), followed by hypertensive disorders (27, 31.0%), sepsis (15, 17.2%), acute fatty liver of pregnancy (11, 12.6%) and amniotic fluid embolism (10, 11.5%). Compared with patients without DIC, those with DIC had higher rates of multiple organ dysfunction syndrome/death (27.6% vs 4.8%, p = 0.000), organ failure (36.8% vs 24.3%, p = 0.029), among which organ failure included acute renal failure (32.2% vs 10.0%, p = 0.000), respiratory failure (16.1% vs 8.6%, p = 0.057), disturbance of consciousness (12.6% vs 2.4%, p = 0.000) and DIC group also had higher rates of massive transfusion (52.9% vs 21.9%, p = 0.000), hysterectomy (32.2% vs 15.7%, p = 0.001), longer ICU (4 days vs 2 days, p = 0.000) and hospital stays (14 days vs 11 days, p = 0.005). DIC and amniotic fluid embolism were independent risk factors for organ failure in patients admitted to the ICU. Postpartum hemorrhage was the leading cause of DIC associated organ failure in obstetrics admitted to the ICU. The control of obstetric bleeding in a timely manner may improve obstetric prognoses.

## Introduction

Disseminated intravascular coagulation (DIC) is a severe complication of obstetric diseases that is characterized by the generation of thrombin with fibrin deposition within the micro- and macrovascular systems with concomitant life-threatening bleeding, and hysterectomy is sometimes performed as a salvage procedure^1^. The incidence of DIC has been reported to be 2.2 to 3.2 per 1000 deliveries in the USA^2^, England and Australia^3^. Although DIC is well known by obstetric clinicians, the underlying diseases of DIC-associated organ failure in critically ill obstetrics patients are not well known. Therefore, a retrospective study was conducted to determine the role of DIC in obstetric conditions in patients admitted to three intensive care units (ICUs) in China.

## Methods

This retrospective cohort study was conducted in the ICUs of three hospitals in Beijing City, China, namely, Peking University Third Hospital, Peking University First Hospital, and Capital Medical University Affiliated Beijing Chao-Yang Hospital. All three hospitals are academic hospitals with critical maternal care centers.

All women in any trimester of pregnancy or within 42 days of delivery who were admitted to any of the three ICUs for obstetric reasons for at least 24 h between January 2008 and December 2016 were included. Women with other hematological conditions and women with a total score comprising the platelet count in peripheral blood plus prothrombin time plus fibrinogen level that was greater than or equal to 2 but less than 5 according to the International Society of Thrombosis and Hemostasis scoring system (ISTH) for deficiencies in fibrin/fibrinogen degradation levels were excluded. DIC cases were identified by scores greater than or equal to 5 based on the ISTH scoring system independent of clinical manifestations such as bleeding or organ failure. d-dimer, a fibrin degradation product (FDP), was used as the fibrin/fibrinogen-related marker for the ISTH criteria.

The following data were recorded: demographic characteristics; diagnosis upon ICU admission; total maximum Sequential Organ Failure Assessment (SOFA) score over the duration of the patient’s stay in the ICU, with the most abnormal value being used for each variable recorded; maternal death; massive transfusion (≥ 10 units), hysterectomy and the level of intervention provided in the ICU (hemofiltration or plasmapheresis). Organ failure was considered to be due to DIC when it appeared to be related solely to DIC or when the manifestations could have been related to the underlying disease but were significantly aggravated by DIC, as shown by a SOFA score greater than or equal to 2. The primary outcome was the change in organ function 24 h after ICU admission, represented with a three-category ordinal scale. It consisted of the following categories: 1, no occurrence of organ failure before discharge or improved organ function with a SOFA score less than or equal to 1; 2, occurrence of single organ failure with a SOFA score greater than or equal to 2; and 3, occurrence of multiple organ dysfunction syndrome (MODS) with no improvement until 24 h after ICU admission or with death before discharge. The durations of hospital stay and ICU stay were recorded as secondary outcomes.

Over 9 years, from 2008 to 2016, 321 cases in women admitted to the three ICU wards for obstetric diseases were reviewed. A total of 23 women with deficient fibrin/fibrinogen degradation levels but with DIC scores greater than or equal to 2 were excluded, and 1 patient with lymphocytoma was excluded. Eighty-seven cases of DIC from the hospital charts of the three tertiary hospitals were identified based on the ISTH DIC scoring system among the 297 women included.

The present study was approved by the Peking University Third Hospital Medical Science Research Ethics Committee, and all information obtained was used only to describe the patient population and for data analysis. The informed consent to participate in the study was waved by the Peking University Third Hospital Medical Science Research Ethics Committee. The methods were carried out in accordance with the relevant guidelines and regulations.

Categorical variables are presented as numbers (%), and continuous variables are presented as the mean ± SD or median (interquartile range) according to their distribution. Normally and nonnormally distributed continuous variables were compared using Student’s t-test and the Mann–Whitney U test, respectively. Categorical variables were compared using the chi-square test or Fisher’s exact test. P values less than or equal to 0.05 were considered significant. The relationships between DIC and the changes in organ function were analyzed using ordinal regression model. All statistical analyses were conducted using the statistical package SPSS, version 26.0 (https://www.lib.pku.edu.cn/portal/cn/zy/software).

### Ethics approval

This study was approved by the ethics committee of Peking University Third Hospital. All data were anonymized, and the requirement for consent was waived.

## Results

### Demographics of patients admitted to the ICU for obstetric disease-related reasons

Within the 9-year study period (2008–2016), 87,850 deliveries occurred. Overall, the mean age of the women admitted to the ICUs was 31 years. The mean gestational age was 34 weeks, and 27.9% of patients were transferred to ICUs directly from another healthcare facility. The median length of ICU stay was 3 days, and the median hospital stay duration was 12 days. The median SOFA and APACHE II scores were 4 and 7, respectively. Over the 9-year study period, the leading diagnoses associated with ICU admission were postpartum hemorrhage (PPH) (164/279, 55.2%) and hypertensive disorders of pregnancy (139/297, 46.8%), followed by sepsis (44/297, 14.8%), acute fatty liver of pregnancy (18/297, 6.1%), and amniotic fluid embolism (13/297, 4.4%) (Table 1).

**Table 1:** The demographics of patients admitted to the ICU for obstetric diseases from 2008 to 2016.

Variables	n = 297 n (%)	DIC (n = 87, %)	Non-DIC (n = 210, %)	p value
Age, y	31 ± 5	31 ± 5	31 ± 5	0.855
Age > 35 y, n (%)	59 (19.9)	16 (18.4)	39 (18.6)	0.971
Nullipara, n (%)	112 (37.7)	38 (43.7)	74 (35.2)	0.172
Gestational age	34 ± 5	33 ± 5	35 ± 5	**0.004**
Cesarean section, n (%)	260 (87.5)	67 (77.0)	193 (91.9)	**0.000**
**Admission type, n (%)**				
Emergency department	110 (37.0)	24 (27.6)	86 (41.0)	**0.030**
Direct transfer from another hospital ^a^	83 (27.9)	32 (36.8)	51 (24.3)	**0.029**
Routine	104 (35.0)	31 (35.6)	73 (34.8)	0.886
SOFA score, median (IQR)	4 (2, 6)	5 (4, 10)	3 (2, 5)	**0.000**
APACHE II score	7 (4, 10)	8 (5, 12)	6 (4, 9)	**0.001**
Massive transfusion (≥ 10 units)	92 (31.0)	46 (52.9)	46 (21.9)	**0.000**
Hysterectomy	61 (20.5)	28 (32.2)	33 (15.7)	**0.001**
Postpartum hemorrhage	60	28	32	1^b^
Postpartum hemorrhage	164 (55.2)	71 (81.6)	93 (44.3)	**0.000**
Placental abruption	8	5	3	0.294^b^
Hysterectomy	0	0	0	
Placenta previa	57	11	46	0.000
Hysterectomy	26	7	19	0.182
Placenta accreta	84	18	66	0.000
Hysterectomy	41	11	30	0.239
Uterine atony	27	17	10	**0.024**
Hysterectomy	11	10	1	**0.037**^c^
Hypertensive disorders	139 (46.8)	27 (31.0)	112 (53.1)	**0.000**
Sepsis	44 (14.8)	15 (17.2)	29 (13.8)	0.449
Acute fatty liver of pregnancy	18 (6.1)	11 (12.6)	7 (3.3)	**0.002**
Amniotic fluid embolism	13 (4.4)	10 (11.5)	3 (1.4)	**0.000**^**c**^

Significant values are in bold.

IQR, interquartile range; SOFA, Sequential Organ Failure Assessment; MODS, multiple organ dysfunction syndrome.

^a^Indicates that patients who were transferred directly to ICU wards from another hospital.

^b^ Represents Fisher’s exact test.

^c^ Represents continuity correction.

### Obstetric DIC incidence

The rate of DIC in obstetric patients admitted to the ICU for obstetric diseases^4^ was 29.3% (87/297), and the obstetric DIC incidence in hospitals was 87 per 87,850 deliveries based on the assumption that obstetric patients with DIC admitted to the ICU represented nearly all the DIC patients in obstetric wards, considering the low SOFA scores of women admitted to the ICU that were reported in our previous study^5^. Compared with those in the non-DIC group, the patients in DIC group had a higher SOFA score (5 vs 3, p = 0.000), higher APACHE II score (8 vs 6, p = 0.001) (Table 1), higher proportion of hysterectomy (28, 32.2% vs 33, 15.7%, p = 0.000), longer ICU stay (4 days vs 2 days, p = 0.000), longer hospital stay (14 days vs 11 days, p = 0.005) (Table 2) and higher proportions of transfer from another healthcare facility (32/87, 36.8% vs 51/210, 24.3%, p = 0.029) but had a lower proportion of cesarean section (67/87, 77.0% vs 193/211, 91.9%, p = 0.000) and a younger gestational age (33 weeks vs 35 weeks, p = 0.004) (Table 1).

**Table 2:** Organ failure of patients with DIC admitted to the ICU for obstetric diseases from 2008 to 2016.

Variables	n = 297 n (%)	DIC (n = 87, %)	Non-DIC (n = 210, %)	p value
**Changes in organ function 24 h after ICU admission**				** < 0.001**^**a**^
1^b^	180 (60.6)	31 (35.6)	149 (71.0)	**0.000**
2^c^	83 (27.9)	32 (36.8)	51 (24.3)	**0.029**
3^d^	34 (11.4)	24 (27.6)^e^	10 (4.7)^f^	**0.000**
ICU length of stay, median (IQR)	3 (1, 5)	4 (2, 7)	2 (1, 4)	**0.000**^**g**^
Hospital length of stay, median (IQR)	12 (8, 17)	14 (10, 22)	11 (8, 16)	**0.005**^**g**^
**ARF after admission**	49 (16.5)	28 (32.2)	21 (10.0)	0.000
Continuous hemofiltration	22	14	8	0.407
Plasmapheresis	15	12	3	**0.032**
Postpartum hemorrhage	29	21	8	**0.009**
Hypertensive disorders	23	10	13	0.069
**Respiratory failure after admission**	32 (10.8)	14 (16.1)	18 (8.6)	**0.057**
Length of ventilation, median (IQR)	2 (1, 5)	3 (1, 16)	2 (1, 4)	0.116^g^
Postpartum hemorrhage	21	14	7	**0.000**^**h**^
Hypertensive disorders	14	4	10	0.127
**Heart failure after admission**	32 (10.8)	10 (11.5)	22 (10.5)	0.797
**Disturbance of consciousness after admission**	16 (5.4)	11 (12.6)	5 (2.4)	**0.000**^**h**^
Postpartum hemorrhage		11	3	0.083^i^
Amniotic fluid embolism		5	2	1^i^
Hypertensive disorders		2	4	**0.036**^**i**^
**Hepatic failure after admission**	15 (5.1)	10 (11.5)	5 (2.4)	**0.003**^**h**^

Significant values are in bold.

IQR, interquartile range; ARF, acute renal failure.

^a^Represents the chi-square for trend value.

^b^1, defined as no organ failure occurring before discharge or improved organ function with a SOFA score less than or equal to 1.

^c^2, defined as single organ function with a SOFA score greater than or equal to 2.

^d^3 indicates that MODS did not improve until 24 h after ICU admission or that death occurred before discharge.

^e^There were four deaths in the DIC group.

^f^There were two deaths in the non-DIC group.

^g^Represents value from the Mann–Whitney U test.

^h^Represents the continuity correction.

^i^Represents the value from Fisher's exact test.

### Diseases associated with DIC

Compared with patients in the non-DIC group, patients in the DIC group had a higher proportion of postpartum hemorrhage (71, 81.6% vs 93, 44.3%, p = 0.000), among which placenta accrete accounted for 20.7% (18/87) and uterine atony accounted for 19.5% (17/87), a higher proportion of acute fatty liver of pregnancy (11, 12.6% vs 7, 3.3%, p = 0.002) and a higher proportion of amniotic fluid embolism (10, 11.5% vs 3, 1.4%, p = 0.000). The DIC group also had a lower proportion of hypertensive disorders of pregnancy (27, 31.0% vs 112, 53.3%, p = 0.000). There were no differences in the rates of sepsis between the DIC and non-DIC groups (15/87, 17.2% vs 29/210, 13.7%, p = 0.449) (Table 1).

### The reasons for hemorrhage-related hysterectomy

Postpartum hemorrhage was the leading cause of hysterectomy in both the DIC group (28/28) and the non-DIC group (32/33). Placenta accreta accounted for 93.8% (30/32) of hemorrhage-related hysterectomies in the non-DIC group, whereas the DIC group had lower proportions of placenta accreta (11/28, 39.3% vs 30/32, 93.8%, p = 0.000)- and placenta previa (7/28, 25.0% vs 19/32, 60.6%, p = 0.007)- associated hysterectomies and a higher proportion of uterine atony (10/28, 35.7% vs 1/32, 3.1%, p = 0.022)- associated hysterectomies. Although similar proportions of hysterectomy were found among patients with uterine atony (11/27)- and placenta accreta (41/84)-associated hemorrhage, patients with placenta accreta were less likely to develop postpartum hemorrhage with DIC (18/84 vs 17/27) (Table 1).

### Changes in organ function associated with DIC

Among 297 patients, 180 did not have obvious organ failure or organ failure improvement (represented by the number 1), 83 had organ failure 24 h after ICU admission (represented by the number 2), and 34 patients had multiple organ failure or died (represented by the number 3). The patients in the DIC group were more likely to develop organ failure (32, 36.8%, vs 51, 24.3%, p = 0.029) and MODS or occurrence of death (24, 27.6% vs 10, 4.7%, p = 0.000) than those in the non-DIC group, among which 4 and 2 deaths occurred in the DIC group and non-DIC group, respectively.

ARF (49, 16.5%) was the most common type of organ failure, with twenty-two and fifteen of the patients had continuous hemofiltration and plasmapheresis, respectively. ARF was followed by respiratory failure (32, 10.8%), heart failure (32, 10.8%), disturbance of consciousness (16, 5.4%) and hepatic failure (15, 5.1%) among all patients (Table 2). There were significant differences in the rates of ARF (28, 32.2% vs 21, 10.0%, p = 0.000), disturbance of consciousness (11, 12.6% vs 5, 2.4%, p = 0.000) and hepatic failure (10, 11.5% vs 5, 2.4%, p = 0.003) between patients with DIC and without DIC, whereas no differences were found in the rates of heart failure (10, 11.5% vs 22, 10.5%, p = 0.797) (Table 2). The rate of respiratory failure in the DIC group was nearly significantly higher than that in the non-DIC group (14, 16.1% vs 18, 8.6%, p = 0.057). Postpartum hemorrhage was the leading cause of DIC-associated ARF (21/28), respiratory failure (14/14) and disturbance of consciousness (11/11) (Table 2).

Eleven patients presented with disturbance of consciousness associated with DIC after ICU admission, among whom 5 patients with amniotic fluid embolism complicated by postpartum hemorrhage fell into a coma and 3 of 5 patients had cardiopulmonary resuscitation, two of whom died. One of 11 patients was diagnosed with eclamptic encephalopathy and postpartum hemorrhage. Another 3 patients were diagnosed with postpartum hemorrhage complicated by hypovolemic shock and 2 patients were diagnosed with acute fatty liver of pregnancy, respectively.

### Variables that were independently associated with change in organ function in patients

Ordinal regression analysis showed that DIC and direct transfer from another hospital had an impact on changes in organ function (p = 0.005, p = 0.044, respectively). Although postpartum hemorrhage had no impact on organ function, it is possible that it induced organ failure by DIC, leading to a higher proportion of postpartum hemorrhage in the DIC group than in the non-DIC group (71, 81.6%, vs 93, 44.3%, p = 0.000) (Table 1). Amniotic fluid embolism was an important cause of organ failure with near significance (p = 0.052). The SOFA score predicted the risk of single organ failure, MODS and death (p = 0.000) (Table 3).

**Table 3:** Variables that were independently associated with changes in organ function in 297 obstetric ICU cases based on the ordinal regression model.

Parameters	β	95% CI of β		OR	p
		Lower	Upper		
DIC	0.510	1.163	2.386	1.665	**0.005**
Cesarean section	− 0.339	0.459	1.107	0.713	0.132
**Admission type (routine as the reference)**					
Direct transfer from another hospital^a^	0.390	1.011	2.157	1.477	**0.044**
Emergency department	0.229	0.849	1.863	1.258	0.253
Postpartum hemorrhage	0.036	0.681	1.578	1.037	0.867
Hypertensive disorders	0.339	0.950	2.074	1.404	0.089
Acute fatty liver of pregnancy	0.586	0.927	3.481	1.796	0.083
Amniotic fluid embolism	0.737	0.993	4.401	2.090	**0.052**
SOFA score	0.137	1.103	1.191	1.146	**0.000**
Gestational age	0.020	0.989	1.052	1.020	0.215

Significant values are in bold.

SOFA, Sequential Organ Failure Assessment.

^a^Indicates that patients who were transferred directly to ICU wards from another hospital.

## Discussion

Prospective studies have shown that the development of DIC in various patient groups is associated with an increase in mortality, even when the cases were non-overt^6–11^. The relationship between DIC and organ function in obstetrics patients admitted to the ICU has rarely been reported, but we found that DIC had an impact on organ failure in our study. Patients with DIC exhibited obvious organ failure requiring organ support, longer ICU and hospital stays, and higher levels of massive transfusion.

The kidney was the most commonly injured organ associated with obstetric DIC in our study. ARF is the leading morbidity associated with maternal mortality^12,13^. The total rate of ARF in our study (49/297, 16.5%) was lower than that in other studies performed in developing countries (24.1% to 61%)^5,13–17^, depending on the definition of ARF). However, the rate was almost the same as that reported by Ferreira DP^14^ when the RIFLE criteria were used in our previous study^5^, but more effort needs to be made to decrease the difference between developed and developing countries (ARF, 5% of obstetric admissions to the ICU)^18^. The frequency of DIC-associated ARF has been reported to be 5% to 31%, depending on the cause and diagnostic criteria of ARF used^19,20^. Studies on the relationship between ARF and DIC in obstetrics are rare and are nearly all case series^19,21–27^. Although Stratta et al.^21,24,28^ found that DIC does not have a significant relationship with the severity of renal failure in patients recovering from kidney conditions and although DIC is not directly related to the morphological changes in the kidney^29^, DIC is associated with maternal mortality^30^; the d-dimer concentration can be used as an independent predictor of PR-AKI^31^, potentially indicating that DIC triggers ARF by provoking the release of cytokines from the endothelium^32^ or interacting with the complementary systems^33,34^, which are important mechanisms of kidney injury^35–37^, but not by thrombosis in the kidney.

Few studies on the relationship between DIC and respiratory failure not involving amniotic fluid embolism have been conducted^38^. Hypertensive disorders have been shown to account for most cases of obstetric acute respiratory distress syndrome (ARDS) or pulmonary edema^39–41^ and DIC, excluding lung viral infections such as COVID-19^42^; however, there have been no studies on the relationship between DIC and ARDS in obstetric hypertensive disorders. Monroe Karetzky reported that noncardiogenic pulmonary edema is the most common cause of respiratory failure in the peripartum period^43^. In our study, postpartum hemorrhage was the most common cause of acute respiratory failure in patients with DIC, which may be attributed to massive infusion-associated acute lung injury. However, obstetric hypertensive disorders were the most common causes of acute respiratory failure in non-DIC patients. Pulmonary edema, a complication of obstetric hypertensive disorders^39,44^, plays a key role.

Although hypertensive disorders are the most common causes of obstetric neurological complications, postpartum hemorrhage complicated by amniotic fluid embolism was the most common cause of DIC-induced neurological complications in our study. The imaging examinations revealed extensive low-density foci indicating amniotic fluid embolism encephalopathy in 2 cases, diffuse brain edema in 5 cases, and subarachnoid hemorrhage in 1 case of 11 cases of DIC-induced neurological complications, all of which indicated cerebral hypoperfusion without timely resuscitation and component transfusion with the proper ratio in a timely manner when DIC-associated hemorrhage occurred.

Direct transfer from another hospital as an independent risk factor for organ failure in patients admitted to the ICU hinted at failure to identify the proper time for transfer to higher level maternal care in lower-level hospitals.

Postpartum hemorrhage was still the leading cause of DIC-induced obstetric organ injury, with a high proportion of hysterectomies in our study. The total rates of hysterectomy (61/87, 850 deliveries) in the three hospitals were nearly the same as that reported by Brian T. Bateman^45^. DIC can develop early, with massive obstetric hemorrhage without other underlying causes; in fact, “pure” obstetric hemorrhage is reportedly the cause of DIC in 25–35% of cases in observational series^46,47^. Although nearly the same proportion of hysterectomies in patients with placenta accreta (41/84)- and uterine atony (11/27)-associated hemorrhage were found, patients with placenta accreta had a lower risk of DIC-associated hemorrhage (18/84 vs 17/27), indicating the benefits of the risk assessment of postpartum hemorrhage before delivery, including establishing an ultrasound-based scoring system for predicting the risk of placenta accreta, assessing the probability of surgical interventions such as embolization of pelvic arteries, hysterectomy, and preparing adequate blood products. On the other hand, DIC is a form of consumptive coagulopathy and is difficult to differentiate from dilutional coagulopathy for untimely transfusion in obstetrics cases with postpartum hemorrhage. Dilutional coagulopathy associated with massive red blood cell transfusion without the replacement of clotting factors and platelets may be the key for the prevention of postpartum hemorrhage. Fixed ratios of packed red blood cells, fresh frozen plasma, and platelets should be used.

DIC is an independent and relatively reliable predictor of organ failure in critically ill obstetrics. To date, whether postpartum hemorrhage is the cause of DIC or the mechanism of DIC-associated obstetric organ failure is not clear. Timely control of obstetric bleeding with methods such as a quick response to maternal hemorrhage with a designated multidisciplinary response team, a staged postpartum hemorrhage protocol including fixed ratios of component transfusion, and even aggressive interventions including the B-Lynch procedure, embolization of pelvic arteries, or ultimately hysterectomy may prevent organ failure in patients with postpartum hemorrhage-associated DIC.
